# Antitumor natural products targeting mitochondrial NADH: ubiquinone oxidoreductase (complex I): a review

**DOI:** 10.3389/fphar.2026.1799383

**Published:** 2026-03-16

**Authors:** Jiaqi Gou, Lingdang Chen, Yuting Xu, Binbin Liao, Yuanmin Shen, Juntao Tai, Jianying Zhang, Aixue Zuo

**Affiliations:** 1 Key Laboratory of Sustainable Utilization of Southern Medicinal Resources, School of Traditional Chinese Medicine, Yunnan University of Traditional Chinese Medicine, Kunming, Yunnan, China; 2 Yunnan University of Traditional Chinese Medicine, School of Basic Medical Sciences, Kunming, Yunnan, China

**Keywords:** antitumor mechanisms, mitochondria, mitochondrial complex I, NADH, ubiquinone oxidoreductase, natural products

## Abstract

Mitochondrial complex I (NADH:ubiquinone oxidoreductase) is a critical hub for bioenergetics and redox signaling. Beyond its canonical role in oxidative phosphorylation and ATP synthesis, complex I regulates the intracellular NADH/NAD^+^ balance and reactive oxygen species (ROS) production, both of which are vital for tumor survival. Consequently, targeting complex I has emerged as a promising therapeutic strategy. Increasing evidence shows that diverse natural products—ranging from alkaloids to annonaceous acetogenins—exert potent antitumor effects by inhibiting complex I. These compounds disrupt mitochondrial function, inducing metabolic stress and cancer cell death. However, a systematic overview linking their chemical structures to specific binding modes and antitumor mechanisms is currently lacking. In this review, we summarize recent advances in natural products targeting mitochondrial complex I. We categorize these agents based on their structural characteristics and discuss their distinct mechanisms, such as acting as “deep tunnel blockers” versus “shallow pocket binders.” This work aims to provide a theoretical foundation for the rational development of novel complex I-targeted antitumor drugs.

## 1 Introduction

Metabolic reprogramming is a fundamental hallmark of cancer. While the Warburg effect—characterized by aerobic glycolysis—was historically considered the dominant metabolic phenotype, recent evidence demonstrates that mitochondria are critical for tumorigenesis and progression. Cancer cells, particularly those exhibiting therapeutic resistance or stem-like properties, maintain functional oxidative phosphorylation (OXPHOS) to meet their elevated biosynthetic and bioenergetic demands (Vasan et al., 2020). Consequently, targeting the mitochondrial electron transport chain has shifted from a neglected area to a focal point of therapeutic intervention.

Mitochondrial complex I (NADH: ubiquinone oxidoreductase) serves as the primary entry point for electrons into the respiratory chain. It plays a dual role in cellular physiology: driving ATP synthesis via proton pumping and regulating the intracellular NAD^+^/NADH redox balance. In the context of cancer, complex I acts as a metabolic hub. Its inhibition not only depletes ATP but also disrupts the regeneration of NAD^+^, which is required for glycolysis and DNA repair. Moreover, complex I is a major site for reactive oxygen species (ROS) production. Dysregulation of this complex can push ROS levels beyond the lethal threshold, triggering selective cancer cell death. Thus, complex I represents a specific vulnerability in metabolically active tumors (Urra et al., 2017).

Natural products have historically provided key structural scaffolds for anti-cancer drug discovery. Owing to their chemical diversity, natural compounds offer unique opportunities to modulate the intricate architecture of complex I. Multiple classes of natural products, including alkaloids and polyphenols, have been reported to inhibit complex I activity. Compared with early inhibitors associated with substantial toxicity (e.g., rotenone), recent efforts have increasingly focused on derivatives and related compounds with improved safety profiles and more favorable therapeutic selectivity (Quintela-Fandino et al., 2020).

In light of the rapid progress in this field, this review summarizes recent advances in natural products targeting mitochondrial NADH:ubiquinone oxidoreductase. Unlike previous reviews that primarily catalogue compound activities, the novelty of our work lies in integrating traditional chemical classifications with emerging structural insights. While we organize these agents by their chemical scaffolds (e.g., alkaloids, terpenoids, quinones), we systematically map them to specific structural binding modes—distinguishing between proposed “deep tunnel blockers” and “shallow pocket binders” based on recent cryo-EM data and structure-activity relationships (SAR). Furthermore, we critically analyze their downstream mechanisms, such as ROS amplification and bioenergetic crisis, while actively addressing the pharmacokinetic and safety challenges limiting their clinical translation. This work aims to provide a theoretical reference and conceptual framework to facilitate the rational development of novel, selective antitumor therapies based on complex I inhibition.

## 2 Literature search strategy and selection criteria

To ensure a rigorous and comprehensive overview, a literature search was conducted using databases including PubMed, Web of Science, and Google Scholar. The search was performed for articles published between 2003 and 2025, using combinations of the following keywords: “Mitochondrial complex I″, “NADH:ubiquinone oxidoreductase”, “natural products”, “inhibitors”, “tumor metabolism”, and “oxidative phosphorylation”. Articles were selected based on their relevance to structural biology (e.g., cryo-EM evidence), *in vitro* mechanistic validation, and *in vivo* antitumor efficacy. Studies lacking definitive evidence linking the natural product’s phenotype directly to complex I modulation were excluded to maintain the mechanistic focus of this review.

## 3 Structure and function of complex I

Mitochondrial complex I is the largest multisubunit assembly in the respiratory chain, consisting of 45 subunits arranged in a characteristic L-shaped architecture. Structurally, it comprises a hydrophilic “peripheral arm” (extending into the mitochondrial matrix) responsible for NADH oxidation, and a hydrophobic “membrane arm” embedded in the inner mitochondrial membrane that mediates proton translocation (Sharma et al., 2009). Functionally, the enzyme catalyzes the transfer of two electrons from NADH to ubiquinone (CoQ) via a flavin mononucleotide (FMN) cofactor and a chain of iron–sulfur clusters. This redox reaction is energetically coupled to the pumping of four protons (H^+^) from the matrix into the intermembrane space, establishing the electrochemical gradient that drives ATP synthesis (Parey et al., 2021).

In eukaryotic cells, the complex is assembled from 14 highly conserved core subunits and approximately 30 supernumerary subunits that regulate stability and assembly. Crucially for drug discovery, the ubiquinone-binding site is located at the interface of the peripheral and membrane arms. This site forms a long, narrow, and deeply buried hydrophobic cavity. Due to its restricted topology, this cavity represents a pharmacological “hotspot” where numerous natural products bind, competitively blocking electron transfer to ubiquinone (Zhu et al., 2016).

**FIGURE 1:**
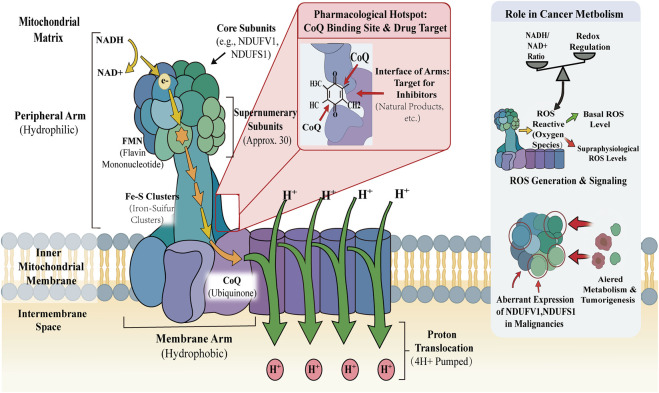
Mitochondrial Complex I - Structure and Function Overview. Blue/Green/Purple: Protein Subunits & Arms:; Yellow/Orange Arrows: Electron Transfer Pathway; Green Arrows: Proton (H+) Translocation; Red Call-out: Pharmacological Target Site; ROS: Reactive Oxygen Species; Fe-S: Iron-Sulfur Cluster; FMN: Flavin Mononucleotide; CoQ: Ubiquinone; NADH:Nicotinamide Adenine Dinucleotide; NDUFV1/NDUFS1:Key Core Subunits implicated in Cancer.

Beyond its bioenergetic function, complex I serves as a critical signaling hub in cancer metabolism. It regulates the intracellular NADH/NAD^+^ redox balance and is a primary source of mitochondrial reactive oxygen species (ROS). Many cancer cells exhibit an increased dependency on complex I to meet the elevated energetic and biosynthetic demands of rapid proliferation. While basal ROS levels support pro-survival signaling (e.g., HIF-1*α* and NF-*κ*B stabilization), supraphysiological ROS bursts induced by complex I dysfunction can exceed the antioxidant capacity of tumor cells, triggering oxidative damage. Consistent with this, aberrant expression of subunits such as NDUFV1 and NDUFS1 has been reported in various malignancies, underscoring the enzyme’s potential as both a biomarker and a therapeutic target (Wallace, 2012) (Figure 1).

## 4 Natural products targeting complex I for antitumor purposes

Natural products remain a fundamental resource for anticancer drug discovery, characterized by their structural diversity and biological relevance. Compared to synthetic libraries, these compounds frequently possess complex stereochemistry and intrinsic affinity for cellular targets (Chen et al., 2023). Recent research has identified a broad spectrum of natural products that function as inhibitors of mitochondrial complex I (NADH: ubiquinone oxidoreductase). By modulating electron transport activity, these agents induce mitochondrial dysfunction and redox stress, thereby contributing to selective tumor cell cytotoxicity (Min et al., 2019). Based on their chemical structures, these inhibitors are categorized into alkaloids, terpenoids, macrolactones, annonaceous acetogenins, flavonoids, coumarins, quinones, and polyphenols (Pourahmad et al., 2014).

**FIGURE 2:**
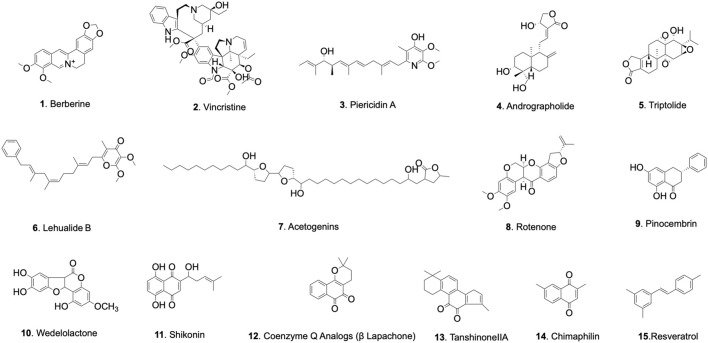
Chemical structures of representative natural products targeting mitochondrial complex I.

The specific chemical characteristics of representative compounds are summarized in Table 1 and their chemical structure are depicted in Figure 2.

**TABLE 1:** Representative natural products targeting mitochondrial complex I.

Chemical class	Compound	Chemical name	Molecular formula	Molecular mass (g/mol)
Alkaloids	Berberine	16,17-dimethoxy-5,7-dioxa-13-azoniapentacyclo [11.8.0.02,10.04,8.015,20] henicosa-1(13),2,4(8),9,14,16,18,20-octaene chloride	C_20_H_18_NO_4_^+^	336.4
Alkaloids	Coptisine	5,7,17,19-tetraoxa-13-azoniahexacyclo [11.11.0.02,10.04,8.015,23.016,20] tetracosa-1(13),2,4(8),9,14,16 (20),21,23-octaene	C_19_H_14_NO_4_^+^	320.3
Alkaloids	Piericidin A	2-[(2E,5E,7E,9R,10R,11E)-10-hydroxy-3,7,9,11-tetramethyltrideca-2,5,7,11-tetraenyl]-5,6-dimethoxy-3-methyl-1H-pyridin-4-one	C_25_H_37_NO_4_	415.6
Diterpenoid	Triptolide	(1S,2S,4S,5S,7R,8R,9S,11S,13S)-8-hydroxy-1-methyl-7-propan-2-yl-3,6,10,16-tetraoxaheptacyclo [11.7.0.02,4.02,9.05,7.09,11.014,18] icos-14 (18)-en-17-one	C_20_H_24_O_6_	360.4
Diterpenoid	Andrographolide	(3E,4S)-3-[2-[(1R,4aS,5R,6R,8aS)-6-hydroxy-5-(hydroxymethyl)-5,8a-dimethyl-2-methylidene-3,4,4a,6,7,8-hexahydro-1H-naphthalen-1-yl] ethylidene]-4-hydroxyoxolan-2-one	C_20_H_30_O_5_	350.4
Macrolactones	Lehualide B	2,3-dimethoxy-5-methyl-6-[(2E,6Z,9E)-3,7,9-trimethyl-11-phenylundeca-2,6,9-trienyl] pyran-4-one	C_28_H_36_O_4_	436.6
Acetogenins	Bullatacin	(2S)-4-[(2R,13R)-2,13-dihydroxy-13-[(2R,5R)-5-[(2R,5R)-5-[(1S)-1-hydroxyundecyl] oxolan-2-yl] oxolan-2-yl] tridecyl]-2-methyl-2H-furan-5-one	C_37_H_66_O_7_	622.9
Flavonoids	Rotenone	(1S,6R,13S)-16,17-dimethoxy-6-prop-1-en-2-yl-2,7,20-trioxapentacyclo [11.8.0.03,11.04,8.014,19] henicosa-3(11),4(8),9,14,16,18-hexaen-12-one	C_23_H_22_O_6_	394.4
Coumarins	Wedelolactone	1,8,9-trihydroxy-3-methoxy-[1] benzofuro [3,2-c] chromen-6-one	C_16_H_10_O_7_	314.25
Quinones	Shikonin	5,8-dihydroxy-2-[(1R)-1-hydroxy-4-methylpent-3-enyl] naphthalene-1,4-dione	C_16_H_16_O_5_	288.29
Quinones	*β*-Lapachone	2,2-dimethyl-3,4-dihydrobenzo [h]chromene-5,6-dione	C_15_H_14_O_3_	242.27
Quinones	Chimaphilin	2,7-dimethylnaphthalene-1,4-dione	C_12_H_10_O_2_	186.21
Quinones	Tanshinone IIA	1,6,6-trimethyl-8,9-dihydro-7H-naphtho [1,2-g][1] benzofuran-10,11-dione	C_19_H_18_O_3_	294.3
Polyphenols	Resveratrol	5-[(E)-2-(4-hydroxyphenyl) ethenyl] benzene-1,3-diol	C_14_H_12_O_3_	228.24

### 4.1 Structural basis and structure-activity relationship (SAR) of inhibitors

While high-resolution cryo-EM structures have mapped the exact binding coordinates for prototypical inhibitors like rotenone and piericidin A, it is important to acknowledge that the precise binding modes for many other natural products discussed herein are putatively assigned based on molecular docking and structure-activity relationship (SAR) studies. A key determinant of binding depth and inhibitory potency is the chemical motif of the compound. For instance, the extended lipophilic tails characteristic of annonaceous acetogenins are essential for penetrating the highly hydrophobic, 30 Å-long ubiquinone-binding tunnels, earning them the designation of “deep tunnel blockers.” Conversely, compounds relying heavily on heterocyclic cores or specific hydrogen-bonding functionalities (e.g., interacting with Tyr144 or His222 of the NDUFS2 subunit) tend to anchor near the entrance, acting as “shallow pocket binders”. Future validation via cryo-EM and mutagenesis is required to definitively confirm these inferred binding modes across all natural product classes.

### 4.2 Alkaloids

#### 4.2.1 Berberine

Berberine is a protoberberine isoquinoline alkaloid isolated from *Coptis chinensis* Franch. Its antitumor efficacy is mechanistically linked to the modulation of mitochondrial complex I function (Wang et al., 2020). Unlike direct enzymatic inhibitors, berberine accumulates within the mitochondrial matrix and activates sirtuin 3 (SIRT3). This activation promotes the deacetylation of the key complex I subunit NDUFS1, leading to the structural disassembly and functional inhibition of the complex (Huang et al., 2023). In gastrointestinal and breast cancer models, this bioenergetic crisis and oxidative stress trigger multiple cell death modalities, including apoptosis, ferroptosis, and autophagy (Mori et al., 2023). Consistently, structural analogs such as coptisine exhibit similar mechanisms, selectively inhibiting complex I to induce metabolic reprogramming in triple-negative breast cancer cells.

However, the biological impact of berberine is not uniform but context-dependent. While it induces lethal oxidative stress in tumor cells, it may activate the KEAP1/Nrf2 signaling pathway in non-malignant models (e.g., PC12 cells), enhancing antioxidant defense and cytoprotection (Zhang et al., 2017). This differential response—exploiting the elevated metabolic demand of cancer cells while sparing normal tissues—underscores the potential of berberine as a selective mitochondrial modulator.

#### 4.2.2 Vinca alkaloids

Vinca alkaloids, exemplified by vincristine and vinblastine, are classical microtubule-destabilizing agents derived from *Catharanthus roseus* (L.) G. Don. While highly effective in chemotherapy, their clinical utility is frequently compromised by severe peripheral neuropathy, a dose-limiting toxicity now mechanistically linked to mitochondrial off-target effects (Chiu et al., 2012). Beyond their canonical action on the cytoskeleton, these alkaloids impair mitochondrial respiration through the inhibition of complexes I and II. In primary sensory neurons, this blockade precipitates an energetic crisis characterized by ATP depletion (Bates and Eastman, 2017).

At the molecular level, this dysfunction involves the peroxynitrite-mediated nitration and subsequent inactivation of mitochondrial manganese superoxide dismutase (MnSOD). The resulting accumulation of ROS exacerbates cellular energy deficits, establishing a vicious cycle of oxidative damage (Svab et al., 2019). Structural modifications can significantly alter this mitochondrial interaction profile. For instance, vinpocetine, a semi-synthetic derivative, modulates mitochondrial respiration to reduce hydrogen peroxide generation, manifesting cytoprotective rather than cytotoxic properties (Lohrasbi et al., 2025).

Consequently, while microtubule interference remains the primary antitumor mode of action for Vinca alkaloids, their interaction with complex I provides the molecular rationale for their neurotoxic limitations. Future structural optimization aimed at minimizing complex I affinity could potentially decouple therapeutic efficacy from these adverse mitochondrial effects.

#### 4.2.3 Piericidin A

Piericidin A is a pyridine alkaloid isolated from *Streptomyces* spp. Due to its structural similarity to the ubiquinone ring system, it functions as a potent competitive inhibitor of Mitochondrial complex I (Tamura et al., 1963). Biochemical and structural analyses have confirmed that Piericidin A binds tightly to the ubiquinone-binding pocket, effectively displacing endogenous ubiquinone. This interaction severs the electron transfer pathway from NADH to the acceptor pool, thereby arresting catalytic activity and disrupting mitochondrial energy transduction (Zhou and Fenical, 2016). In cellular contexts, the blockade imposed by Piericidin A leads to a highly reduced state of the flavin mononucleotide (FMN) site. This results in significant electron leakage and the rapid production of reactive oxygen species (ROS) (Ahmed et al., 2021). However, the therapeutic window of Piericidin A is narrow. Studies involving metabolic stressors, such as isoniazid, indicate that Piericidin A-mediated complex I inhibition can precipitate severe ATP depletion and hepatotoxicity (Choi et al., 2011). Consequently, while its pronounced systemic toxicity limits its direct clinical application, Piericidin A remains an indispensable chemical probe for mapping the inhibitor-binding interface and dissecting the mechanistic role of complex I in tumor metabolism.

### 4.3 Diterpenoid

#### 4.3.1 Andrographolide

Andrographolide is a labdane diterpenoid isolated from *Andrographis paniculata (Burm.f.) Wall. ex Nees*, noted for its diverse pharmacological activities. Unlike classical inhibitors that disrupt electron transport, emerging evidence suggests that andrographolide functions primarily as a modulator of mitochondrial respiratory integrity (Geng et al., 2019).

In a rat model of nicotine-induced brain mitochondrial oxidative stress, andrographolide co-administration mitigated nicotine-mediated inhibition of mitochondrial electron transport chain complexes I–III, reduced nitric oxide and superoxide anion generation, and restored antioxidant defenses, including catalase and glutathione-dependent enzymes (Das et al., 2009). These findings indicate that, rather than blocking electron flow, andrographolide may stabilize the respiratory chain components to maintain redox homeostasis.

However, the precise molecular interaction between andrographolide and complex I remains to be fully elucidated. Current data do not support a mechanism involving direct, competitive binding to the NADH or ubiquinone sites characteristic of classical inhibitors. Consequently, further structural and biochemical studies are required to determine whether andrographolide exerts its effects through allosteric modulation or indirect signaling pathways, and to clarify how this mitochondrial regulation contributes to its established antitumor phenotype (Murai et al., 2009). This suggests that Andrographolide acts as a modulator rather than a pure inhibitor. In cancer cells, however, this modulation may disrupt the precarious redox balance, acting distinctly from its cytoprotective role in normal tissues.

#### 4.3.2 Triptolide

Triptolide, a diterpenoid epoxide derived from *Tripterygium wilfordii Hook. f.* severely compromises mitochondrial bioenergetic homeostasis. Its antitumor efficacy is fundamentally linked to the impairment of complex I function, which precipitates a bioenergetic crisis characterized by ATP depletion and the dissipation of the mitochondrial membrane potential (Δ*Ψ*m) (Meng et al., 2014).

In non-small cell lung and ovarian cancer phenotypes, this respiratory blockade drives a lethal surge in reactive oxygen species (ROS). Crucially, this oxidative stress functions as a signaling modulator rather than merely a cytotoxic byproduct: elevated ROS levels suppress the NF-*κ*B pathway and downregulate anti-apoptotic proteins such as Bcl-2, thereby committing cells to caspase-3-dependent apoptosis (Liu et al., 2025).

Beyond direct bioenergetic suppression, triptolide modulates complex I through post-translational mechanisms. In p53-deficient cancer cells, it suppresses the expression of the mitochondrial deacetylase sirtuin 3 (SIRT3). The consequent hyperacetylation of respiratory complexes I and II compromises their enzymatic efficiency, further exacerbating metabolic stress (Kumar et al., 2016). This multifaceted disruption of mitochondrial homeostasis sensitizes resistant tumors to chemotherapeutic agents like cisplatin. However, the dependence on p53 status indicates that triptolide acts as a context-specific mitochondrial toxin, necessitating precise stratification in therapeutic applications (Wang et al., 2015).

### 4.4 Macrolactones

#### 4.4.1 Lehualide B

Lehualide B, a marine-derived macrolactone, represents a distinct structural class of mitochondrial inhibitors. Biochemical characterization has established that Lehualide B targets the mitochondrial electron transport chain, specifically inhibiting complex I (NADH:ubiquinone oxidoreductase). Its inhibitory potency against the enzyme is in the nanomolar range, comparable to classical inhibitors such as piericidin A and rotenone.

The compound displays context-dependent cytotoxicity, exhibiting exceptional potency against human multiple myeloma cells compared to solid tumor lines such as breast cancer. Quantitatively, its cytotoxic activity against myeloma cells is approximately two- to three-fold higher than that observed in breast cancer models. This selectivity likely reflects the differential metabolic dependencies of these tumor types: plasma cell malignancies often exhibit heightened reliance on oxidative phosphorylation and possess limited metabolic plasticity to compensate for respiratory blockade.

Structure–activity relationship (SAR) analyses have delineated the molecular determinants of this activity. Both the *γ*-pyranone head group and the conjugated polyene side chain are identified as critical pharmacophores. Analogs lacking these moieties exhibit markedly reduced inhibitory efficacy, indicating that these structural features are essential for precise docking within the ubiquinone-binding pocket of complex I (Jeso et al., 2013). Collectively, these data position Lehualide B as a promising lead scaffold for developing selective inhibitors targeting OXPHOS-dependent malignancies.

### 4.5 Annonaceous acetogenins

Annonaceous acetogenins represent a unique class of polyketide-derived natural products isolated from the Annonaceae family. Structurally, they are characterized by a long aliphatic chain containing one or more tetrahydrofuran (THF) rings and a terminal *γ*-lactone moiety. Compounds such as bullatacin, asimicin, and squamocin are recognized as some of the most potent mitochondrial complex I inhibitors known, often exhibiting cytotoxicity in the sub-nanomolar range (Jacobo-Herrera et al., 2019).

Recent cryo-electron microscopy (cryo-EM) studies have fundamentally redefined the mechanism of these compounds, classifying them as Deep Tunnel Blockers. Unlike inhibitors that bind superficially, the hydrophobic tail of acetogenins penetrates the full length of the ubiquinone-binding tunnel at the NDUFS2/NDUFS7 interface (Grba et al., 2022). This deep steric occlusion acts as a structural wedge, completely blocking ubiquinone access and electron transfer.

The physiological consequences of this “deep blockade” are twofold. First, it causes a severe bioenergetic collapse due to ATP depletion. Second, and perhaps more critically for therapy, it triggers massive electron back-pressure, resulting in a burst of superoxide generation. This mechanism has been linked to the induction of ferroptosis, particularly in drug-resistant tumors that are refractory to apoptosis (Lima et al., 2022).

However, the exceptional potency and lipophilicity of acetogenins present a double-edged sword. Their ability to cross the blood-brain barrier has been associated with neurotoxicity (specifically atypical parkinsonism) due to chronic inhibition of neuronal respiration (Fallon Adido et al., 2023). Consequently, current translational efforts focus on structural optimization—such as the development of antibody-drug conjugates (ADCs) or hydrophilic analogs—to harness their “deep tunnel” inhibitory potential while improving the therapeutic index.

### 4.6 Flavonoids

#### 4.6.1 Rotenone

Rotenone, a naturally occurring isoflavonoid, serves as the canonical inhibitor of mitochondrial complex I. Structural insights derived from cryo-EM have defined Rotenone as a prototypical Shallow Pocket Binder. In stark contrast to the “deep tunnel” penetration of acetogenins, Rotenone occupies only the entrance of the ubiquinone-binding cavity (Q-site) without traversing the full length of the hydrophobic tunnel. This specific binding mode effectively prevents ubiquinone access, thereby severing electron transfer from the iron-sulfur clusters to the CoQ pool (Heinz et al., 2017).

The bioenergetic consequences of this “shallow blockade” are profound. Rotenone treatment precipitates a rapid collapse of the mitochondrial membrane potential and ATP depletion. Furthermore, the interruption of electron flow at this proximal site promotes significant electron leakage, generating a burst of superoxide anions (O_2_^−^) (Li et al., 2003). In oncology models, this mechanism is particularly effective against multidrug-resistant (MDR) phenotypes, such as SW480 cells. These resistant populations, often refractory to standard chemotherapy, remain vulnerable to Rotenone-induced, ROS-mediated apoptosis, highlighting the potential of metabolic intervention to overcome drug resistance (Ozay et al., 2018).

However, the clinical translation of Rotenone is precluded by its severe systemic toxicity and neurotoxicity (specifically its utility in inducing Parkinson’s disease models). Consequently, Rotenone functions not as a therapeutic candidate, but as a vital proof-of-concept agent. It validates the “shallow pocket” of complex I as a druggable vulnerability, guiding the design of synthetic analogs with optimized safety profiles (Heo et al., 2022).

#### 4.6.2 Pinocembrin

Pinocembrin, a flavanone abundant in propolis and honey, modulates mitochondrial bioenergetics with distinct outcomes depending on the biological context. Mechanistic insights from eukaryotic models, specifically *Penicillium italicum*, reveal that pinocembrin acts as a dual inhibitor of mitochondrial complexes I and V. This enzymatic blockade precipitates a severe bioenergetic failure, characterized by ATP depletion, mitochondrial membrane depolarization, and the opening of the permeability transition pore (mPTP), ultimately driving programmed cell death (Sangweni et al., 2020).

In contrast to this cytotoxic profile, pinocembrin exhibits cytoprotective properties in mammalian neuronal models. In SH-SY5Y cells challenged with the oxidative toxin paraquat, pinocembrin preserves mitochondrial function rather than disrupting it. These protective effects are mediated by the upregulation of antioxidant defenses via the ERK1/2–Nrf2 signaling axis, which suppresses ROS-induced Bax activation and cytochrome c release (Kumar et al., 2007). This functional dichotomy suggests that pinocembrin possesses a complex pharmacological profile. While its ability to target the respiratory chain is established in lower eukaryotes, its potential as an anticancer agent hinges on translating this mitochondrial toxicity to tumor cells without compromising neuronal integrity. Future studies must specifically validate whether the complex I inhibitory mechanism observed in fungal models can be effectively recapitulated in human malignancies (Yang et al., 2018).

### 4.7 Coumarin compounds

#### 4.7.1 Wedelolactone

Wedelolactone, a coumestan derivative naturally occurring in Asteraceae species such as *Eclipta prostrata*, functions as a pleiotropic metabolic disruptor. Mechanistic studies reveal that its antitumor efficacy stems from a dual metabolic blockade targeting both mitochondrial respiration and aerobic glycolysis (Sarveswaran et al., 2016).

Primarily, wedelolactone acts as a direct inhibitor of mitochondrial complex I. This enzymatic blockade restricts electron transfer, elevating the intracellular NADH/NAD^+^ ratio and impeding ATP synthesis. Concurrently, the interruption of electron flow triggers a surge in reactive oxygen species (ROS). This oxidative stress initiates apoptotic cascades, evidenced by caspase-3 activation and PARP cleavage (Li et al., 2020).

Crucially, wedelolactone prevents the metabolic compensation often seen with mitochondrial inhibitors. It simultaneously suppresses the Warburg effect by downregulating key glycolytic enzymes, including Hexokinase 2 (HK2) and Lactate Dehydrogenase A (LDHA) (Kučírková et al., 2018). This creates a theoretical paradox: while NADH accumulation typically stabilizes the pro-survival factor HIF-1*α* (by inhibiting prolyl hydroxylases), the profound ROS burst induced by wedelolactone overrides this protective signal, shifting the cell fate toward apoptosis (Benes et al., 2012).

Beyond metabolism, this compound amplifies cytotoxic stress by suppressing survival signaling axes, including NF-*κ*B and AKT/mTOR (Du et al., 2023). Through this concerted disruption of bioenergetics and redox homeostasis, wedelolactone effectively limits tumor plasticity. However, rigorous *in vivo* pharmacokinetic assessments are still required to determine whether clinically relevant concentrations can be achieved without systemic toxicity.

### 4.8 Quinones

Quinones constitute a class of redox-active natural scaffolds characterized by their ability to undergo reversible reduction-oxidation cycles. This chemical property allows them to act as effective interceptors of electron transport within the mitochondria. Many quinone derivatives selectively disrupt tumor bioenergetics by targeting respiratory chain complex I, catalyzing the generation of reactive oxygen species (ROS) via futile redox cycling, and precipitating a collapse of redox homeostasis.

#### 4.8.1 Shikonin

Shikonin, a naphthoquinone derivative isolated from *Lithospermum erythrorhizon*, exerts selective antitumor effects through a multifaceted disruption of mitochondrial physiology (Wang et al., 2021). Its primary mechanism involves the inhibition of complex I activity, which stalls NADH oxidation. This blockade not only impairs ATP synthesis but also promotes electron leakage, resulting in a surge of superoxide anions (O_2_^−^) (Valipour, 2022).

Concomitantly, shikonin dismantles the cellular antioxidant defense system by suppressing glutathione (GSH) synthesis. This dual-action mechanism—simultaneously generating ROS while depleting scavenging capacity—imposes overwhelming oxidative stress on tumor cells, triggering intrinsic apoptosis (Chen et al., 2021). Furthermore, the resultant depletion of the NAD^+^ pool inhibits NAD^+^-dependent enzymes, including SIRT1 and PARP1. This metabolic-epigenetic crosstalk compromises DNA repair mechanisms, thereby sensitizing hypoxic tumor cells to DNA-damaging chemotherapies (Lew et al., 2025).

Beyond immediate cytotoxicity, shikonin-induced mitochondrial stress remodels downstream oncogenic signaling. By modulating the NF-*κ*B/STAT3 axis, shikonin suppresses epithelial–mesenchymal transition (EMT) and downregulates the immune checkpoint ligand PD-L1 (Wang et al., 2021; Valipour, 2022). These findings suggest that shikonin acts not merely as a mitochondrial toxin, but as a systemic regulator that couples metabolic stress to the suppression of metastasis and immune evasion. Future translational efforts should focus on optimizing delivery systems to improve bioavailability and exploring rational combinations that exploit these metabolic vulnerabilities.

#### 4.8.2 Coenzyme Q analogs (*β*-lapachone)

*β*-Lapachone, a naturally occurring naphthoquinone, functions through a mechanism distinct from direct complex I inhibitors. It acts as a bioactivatable substrate for the cytosolic enzyme NAD(P)H:quinone oxidoreductase 1 (NQO1). Unlike standard quinone detoxification, the interaction between *β*-lapachone and NQO1 establishes a futile redox cycle. The enzyme reduces the quinone to an unstable hydroquinone, which spontaneously auto-oxidizes back to the parent compound using molecular oxygen (Tumbath et al., 2024). This continuous cycling imposes a severe bioenergetic burden: it rapidly consumes NAD(P)H equivalents while generating stoichiometric amounts of superoxide anions (O_2_^−^). Within minutes, this process precipitates a massive accumulation of ROS. The sustained oxidation of the intracellular NAD(P)H pool—often exceeding 60% depletion—creates a profound redox imbalance (Li Y. et al., 2023).

Concurrently, ROS-induced DNA strand breaks trigger the hyperactivation of poly (ADP-ribose) polymerase 1 (PARP1). PARP1 consumes the remaining NAD^+^ pool for DNA repair, causing a severe bioenergetic collapse where ATP synthesis halts due to substrate deprivation. This unique sequence of events drives a distinct form of cell death known as NQO1-dependent programmed necrosis (Huang et al., 2012). Beyond bioenergetic failure, *β*-lapachone actively modulates iron homeostasis. It promotes NCOA4-mediated ferritinophagy via JNK signaling, releasing labile iron that fuels Fenton reactions. When coupled with the depletion of glutathione (GSH) and inhibition of GPX4, this lipid peroxidation drives a subset of cells toward ferroptosis (Becher et al., 2025).

The therapeutic window of *β*-lapachone relies on the differential expression of NQO1. This enzyme is frequently overexpressed (5- to 200-fold) in solid tumors, such as non-small cell lung and pancreatic cancers, compared to adjacent normal tissue. This tumor-restricted bioactivation underpins its synergistic potential with metabolic modulators and ionizing radiation (Song et al., 2008).

#### 4.8.3 Chimaphilin

Chimaphilin, a naphthoquinone constituent of the Ericaceae family, exhibits a pharmacological profile characterized by the dual targeting of growth factor signaling and mitochondrial bioenergetics. While initially identified as an inhibitor of the Insulin-like Growth Factor I Receptor (IGF-IR) tyrosine kinase, subsequent studies indicate that its cytotoxicity involves a profound disruption of mitochondrial homeostasis (Daqian et al., 2015). In breast cancer models (MCF-7), chimaphilin triggers the intrinsic mitochondrial apoptotic pathway. This process is marked by a surge in reactive oxygen species (ROS), the dissipation of the mitochondrial membrane potential (Δ*Ψ*m), and a shift in the Bcl-2/Bad ratio, culminating in the activation of the caspase-9/3 cascade (Chen et al., 2016). This mitochondrial dysfunction is likely reinforced by the upstream inhibition of IGF-IR, which suppresses the PI3K/Akt survival axis.

Beyond direct cytotoxicity, chimaphilin modulates the metastatic phenotype. By inhibiting TGF-*β*1-induced signaling (involving PI3K/Akt, ERK1/2, and Smad pathways), it suppresses epithelial–mesenchymal transition (EMT). This results in the molecular reversal of the invasive phenotype—upregulating E-cadherin while downregulating mesenchymal markers like Vimentin and Snail1 (Ding et al., 2025).

In the context of drug resistance, chimaphilin functions as a chemosensitizer. In multidrug-resistant osteosarcoma, it restores sensitivity to doxorubicin. This effect is attributed to the convergent suppression of IGF-IR signaling and the impairment of cellular energy metabolism (Ali et al., 2024). These findings suggest that chimaphilin exerts antitumor activity by coupling signal transduction inhibition with oxidative stress-mediated mitochondrial apoptosis. Future investigations should elucidate the precise molecular interplay between these two mechanisms to optimize combination therapies (Li et al., 2016).

#### 4.8.4 Tanshinone IIA

Tanshinone IIA (Tan IIA), a major bioactive phenanthrenequinone from *Salvia miltiorrhiza*, exerts pronounced antitumor effects, with a particular efficacy profile in hepatocellular carcinoma (HCC). Mechanistically, Tan IIA orchestrates a dual cell death program involving both apoptosis and necroptosis. This is achieved by downregulating the FLICE-inhibitory protein short form (FLIPS), which acts as a molecular switch to promote Caspase-8 activation, RIP1/RIP3 phosphorylation, and MLKL-mediated necroptosis (Li H. et al., 2023).

At the organelle level, Tan IIA disrupts homeostasis to reinforce lethality. It triggers mitochondrial dysfunction via the elevation of intracellular ROS, which activates the p38 MAPK axis and induces cell-cycle arrest at the S and G2/M phases (Lee et al., 2010). Simultaneously, it provokes endoplasmic reticulum (ER) stress, evidenced by the upregulation of stress markers such as calreticulin and GADD153. This organelle crosstalk shifts the Bax/Bcl-2 ratio, reinforcing the commitment to apoptosis.

Beyond canonical signaling, recent epigenetic profiling reveals that Tan IIA targets the RNA methyltransferase METTL3. By downregulating METTL3-mediated m^6^A modification, it destabilizes the mRNA of the oncogene TRIB3. This transcriptional suppression effectively inhibits tumor proliferation and stem-like properties (Naz et al., 2020).

In the context of therapeutic combinations, Tan IIA demonstrates significant potential. *In vivo* studies confirm its ability to suppress tumor growth and vascularization (indicated by reduced CD31 levels) (Xu et al., 2024). Furthermore, when combined with standard agents like sorafenib, Tan IIA synergizes to inhibit metastasis-related epithelial–mesenchymal transition (EMT) signaling. This suggests that incorporating Tan IIA could offer a strategy to enhance cytotoxicity and overcome resistance to established liver cancer therapies.

### 4.9 Polyphenols

#### 4.9.1 Resveratrol

Resveratrol is a naturally occurring polyphenol whose antitumor activity is intrinsically linked to the modulation of mitochondrial bioenergetics. Biochemical and structural analyses have established that resveratrol targets the ubiquinone-binding pocket of mitochondrial complex I. This interaction is characterized by a distinct hormetic profile: whereas physiological concentrations (<5 μM) may mildly stimulate electron transfer, pharmacological concentrations (e.g., 50 μM) exert a potent inhibitory effect, blocking NADH oxidation and reducing ATP synthesis (Gueguen et al., 2015).

In the context of tumor metabolism, this high-dose inhibition imposes a state of metabolic stress. The blockade of complex I necessitates a compensatory shift toward glycolysis. Concomitant with this metabolic reprogramming, the interruption of the electron transport chain exacerbates electron leakage, driving the generation of superoxide anions (O_2_^−^) and disrupting intracellular redox homeostasis (Kursvietiene et al., 2023).

Beyond direct physical interaction, resveratrol modulates mitochondrial function through the NAD^+^-dependent deacetylase SIRT1. Although complex I inhibition alters the NAD^+^/NADH pool, resveratrol-mediated SIRT1 activation appears to reinforce the suppression of mitochondrial respiration, particularly in breast cancer phenotypes. This SIRT1-mitochondria axis contributes to both cytotoxicity and the induction of cellular differentiation (Madreiter-Sokolowski et al., 2016).

Downstream of these bioenergetic perturbations, resveratrol triggers the mitochondrial apoptotic cascade. By upregulating the pro-apoptotic protein Bax, it induces mitochondrial outer membrane permeabilization (MOMP) and cytochrome c release, ultimately activating caspase-3 (Gueguen et al., 2015). Despite these multi-targeted mechanisms, the clinical utility of resveratrol is constrained by its rapid metabolism and poor bioavailability. Consequently, future translational efforts must prioritize structural optimization and advanced delivery systems to enhance its pharmacokinetic profile.

To synthesize the translational landscape of these findings, representative *in vivo* studies demonstrating the antitumor efficacy of natural products targeting complex I are summarized in Table 2.

**TABLE 2:** *In vivo* antitumor effects and mechanisms of natural products targeting mitochondrial complex I.

Chemical class	Compound	Proposed binding mode/Efficacy (IC50)	Tumor model (n/group)	Dose, route and schedule	Endpoints and proposed downstream mechanism	Toxicity findings/Safety profile	References
Alkaloids	Berberine	Modulator (indirectly via SIRT3) IC50: ∼9.7–17.2 μM	Colorectal & AML xenografts. Vehicle-controlled, n = 5/group	50–100 mg/kg; p.o., daily for 21–28 days	Endpoint: Tumor regression was observed. Mechanism: SIRT3-mediated disassembly of complex I, subsequently inducing metabolic reprogramming	The compound exhibited a favorable tolerability profile, with no significant body weight loss observed during the dosing period	Wang et al. (2020), Huang et al. (2023), Mori et al. (2023)
Diterpenoid	Triptolide	Direct inhibitor IC50: ∼10–50 nM	Pancreatic and NSCLC xenografts. Vehicle-controlled, n = 5–6/group	0.2–0.5 mg/kg; i.p., twice weekly	Endpoint: Exhibits potent growth inhibition. Mechanism: Hyperacetylation occurs in respiratory complexes, coupled with ROS-mediated apoptosis	Potential hepatotoxicity and nephrotoxicity at higher doses	Meng et al. (2014), Liu et al. (2025), Kumar et al. (2016)
Macrolactones	Lehualide B	Shallow pocket binder IC50: ∼230–830 nM	Multiple myeloma models. *In vitro* validation primarily	N/A (requires further *in vivo* PK evaluation)	Mechanism: Leads to severe bioenergetic failure; demonstrates highly selective cytotoxicity against OXPHOS-dependent plasma cell malignancies	Based on preclinical cellular model studies, the drug possesses an optimal therapeutic window	Jeso et al. (2013)
Acetogenins	Bullatacin	Deep tunnel blocker IC50: Sub-nanomolar (<1 nM)	Drug-resistant solid tumors. Vehicle-controlled, n = 6/group	15–50 μg/kg; i.p., every other day	Mechanism: Complete occlusion of the Q-site in the electron transport chain, triggering a massive superoxide burst, which in turn induces ferroptosis and ATP depletion	Due to its ability to penetrate the blood-brain barrier, the compound presents a high risk of neurotoxicity, primarily manifesting as atypical parkinsonism	Jacobo-Herrera et al. (2019), Grba et al. (2022)
Coumarins	Wedelolactone	Direct inhibitor IC50: ∼10–25 μM	Hepatocellular carcinoma xenografts. Vehicle-controlled, n = 5/group	20–50 mg/kg; p.o., daily	Endpoint: Reduced tumor burden. Mechanism: Achieved through dual suppression of OXPHOS and aerobic glycolysis, accompanied by the inhibition of the AKT/mTOR signaling pathway	The toxicological response was mild, and no severe organ toxicity was detected within the effective therapeutic dose range	Sarveswaran et al. (2016), Kučírková et al. (2018), Li et al. (2020)
Quinones	Shikonin	Direct inhibitor IC50: ∼2–5 μM	Lung and breast cancer models. Vehicle-controlled, n = 5–6/group	2–10 mg/kg; i.p., every other day	Mechanism: Disrupts NADH/NAD^+^ balance, concurrently inducing a massive ROS burst and GSH depletion, ultimately leading to necroptosis	Transient body weight reduction was observed, but the overall systemic toxicity level remains low	Wang et al. (2021), Valipour (2022), Chen et al. (2021)
Quinones	β-Lapachone	Modulator (futile redox cycling via NQO1) IC50: ∼1–3 μM (in NQO1+ cells)	NQO1-overexpressing solid tumors. Vehicle-controlled, n = 6–10/group	10–20 mg/kg; i.v., every other day	Mechanism: NQO1-dependently causes rapid NAD(P)H depletion, followed by PARP1 hyperactivation, thereby mediating programmed necrosis	It demonstrates tumor selectivity; the risk of methemoglobinemia was effectively mitigated through nanoparticle delivery	Tumbath et al. (2024), Huang et al. (2012), Becher et al. (2025)
Polyphenols	Resveratrol	Shallow pocket binder IC50: >50 μM (high dose)	Breast and colon cancer xenografts. Vehicle-controlled, n = 5/group	20–100 mg/kg; p.o., daily	Mechanism: Exhibits concentration-dependent blockade of NADH oxidation; proceeds via bax-mediated mitochondrial outer membrane permeabilization (MOMP)	The compound is rapidly metabolized and exhibits poor bioavailability, consequently resulting in very low systemic toxicity	Gueguen et al. (2015), Kursvietiene et al. (2023)

Abbreviations Guide: *p.o*. (oral administration); *i.p*. (intraperitoneal); *i.v*. (intravenous).

## 5 Mechanistic integration of antitumor effects induced by complex I inhibition

**FIGURE 3:**
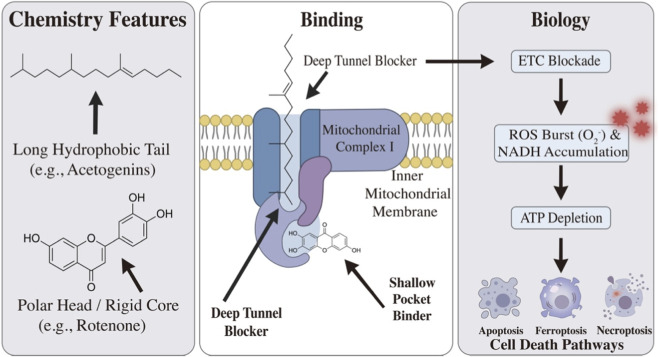
Schematic illustration of the structural characteristics, binding modes, and downstream biological effects of natural products targeting mitochondrial complex I. (Left) Chemical features of representative Complex I inhibitors, categorized into compounds with a long hydrophobic tail (e.g., acetogenins) and those with a polar head or rigid core (e.g., rotenone and flavonoids). (Middle) Distinct binding mechanisms at the ubiquinone reduction site of Complex I, which is embedded in the inner mitochondrial membrane (IMM). Long-chain compounds act as deep tunnel blockers, whereas rigid/heterocyclic compounds function as shallow pocket binders. (Right) The intracellular cascade triggered by Complex I inhibition. The blockade of the electron transport chain (ETC.) leads to a substantial burst of reactive oxygen species (ROS, $O_2^-$) and NADH accumulation. The subsequent ATP depletion ultimately drives the cells toward specific programmed death pathways, including apoptosis, ferroptosis, or necroptosis.

While individual natural products exhibit distinct pharmacological profiles, their engagement with mitochondrial complex I precipitates a convergent sequence of bioenergetic and signaling events. This section integrates these downstream consequences into a unified mechanistic framework, highlighting how respiratory blockade translates into metabolic stress. As illustrated in Figure 3, the specific chemical motifs of these inhibitors dictate their spatial binding modes (deep versus shallow), which in turn translates into profound metabolic stress.

### 5.1 The ROS-signaling nexus: from oxidative stress to cell death

Mitochondrial complex I is not merely an electron pump but a dominant regulator of intracellular redox signaling. Its inhibition interrupts the electron transfer chain, creating a site of electron leakage that catalyzes the excessive generation of superoxide anions (O_2_^−^). Unlike transient physiological ROS, this sustained oxidative burst exceeds the antioxidant buffering capacity of cancer cells. Significantly, this oxidative stress functions as a signal transduction modulator. Elevated ROS actively modulates kinase cascades: it activates stress sensors such as AMPK and JNK while simultaneously suppressing the pro-survival mTORC1 axis. Furthermore, ROS-induced depolarization of the mitochondrial membrane acts as the trigger for cytochrome c release. This creates a feed-forward loop where bioenergetic impairment amplifies oxidative damage, promoting intrinsic apoptosis or, under conditions of severe mitochondrial damage, necroptosis or ferroptosis.

### 5.2 The NAD^+^/NADH axis: precipitating acute metabolic failure

Beyond ATP depletion, the blockade of complex I induces a profound redox imbalance by halting NADH oxidation. This leads to an accumulation of NADH and a concurrent depletion of the NAD^+^ pool. Since NAD^+^ is an obligate cofactor for sirtuins (SIRTs) and PARPs, its depletion impairs key survival mechanisms, including DNA repair and mitochondrial quality control (mitophagy). Crucially, this redox imbalance creates a significant metabolic constraint. High cytosolic NADH levels inhibit the glycolytic pathway because the regeneration of NAD^+^ is required for glycolytic enzymes to function. Consequently, cancer cells face a dual restriction: they are unable to generate ATP via respiration due to complex I inhibition, and they cannot sufficiently upregulate glycolysis to compensate due to the depletion of the NAD^+^ pool. This combined inhibition leads to a profound suppression of cellular metabolism.

### 5.3 Biological basis for therapeutic selectivity

A critical challenge in mitochondrial pharmacology is distinguishing malignant targets from healthy tissues. The potential for therapeutic selectivity with complex I inhibitors is based on the differential metabolic characteristics of cancer cells compared to normal cells. This selectivity relies on three key physiological factors:

First, the difference in oxidative stress thresholds. Cancer cells often operate with higher basal levels of ROS and are closer to their antioxidant capacity limit compared to normal cells. Therefore, the additional ROS generated by complex I inhibition is more likely to push tumor cells beyond their tolerance threshold, whereas normal cells typically possess sufficient antioxidant reserves to buffer this stress.

Second, metabolic inflexibility. Unlike healthy tissues, which can dynamically switch fuel sources to maintain homeostasis, many tumors—particularly those with stem-like properties—exhibit a rigid dependence on oxidative phosphorylation (OXPHOS). This reliance renders them less capable of adapting to respiratory blockade.

Third, the requirement for NAD^+^ turnover. Rapidly proliferating cancer cells have a significantly higher demand for NAD^+^ to support biosynthesis and DNA repair. Complex I inhibition selectively disrupts this supply, thereby exerting a preferential inhibitory effect on the metabolically active tumor microenvironment while sparing quiescent normal cells.

### 5.4 Challenges in selectivity, toxicity, and metabolic resistance

Despite the biological rationale for targeting complex I, realizing a viable therapeutic window remains a profound challenge. Because complex I is essential for basal respiration in normal tissues, systemic non-selective inhibition poses significant toxicity risks, most notably neurotoxicity (e.g., atypical parkinsonism observed with prolonged acetogenin or rotenone exposure) and potential cardiotoxicity. To mitigate these off-target effects, emerging strategies focus on exploiting the tumor’s hyperactive state via prodrugs (e.g., NQO1-bioactivatable β-lapachone) or utilizing mitochondria-targeted delivery systems (such as triphenylphosphonium (TPP) conjugation) to restrict exposure to malignant tissues. Furthermore, tumor metabolic plasticity can blunt the efficacy of complex I inhibitors. Upon acute OXPHOS blockade, cancer cells frequently upregulate aerobic glycolysis (the Warburg effect) or rely on alternative fuel sources like fatty acid oxidation (FAO) as a compensatory survival mechanism. Therefore, future clinical translation will likely depend on rational combination therapies—for example, pairing natural complex I inhibitors with glycolytic inhibitors (e.g., 2-Deoxyglucose) or immune checkpoint modulators to trap tumors in a metabolic dead-end.

## 6 Conclusion

Mitochondrial complex I is increasingly recognized as a significant therapeutic target in oncology. This review summarizes how structurally diverse natural products inhibit this enzyme, thereby suppressing bioenergetic function, altering the NAD^+^/NADH redox balance, and inducing ROS-mediated signaling. From the binding modes of flavonoids to the redox cycling of quinones, current evidence demonstrates that targeting mitochondrial respiration is a feasible strategy to impair tumor metabolism.

However, the clinical translation of these natural products faces significant challenges. A primary limitation is the pharmacokinetic profile, as many candidates exhibit poor solubility, rapid metabolism, or limited tumor accumulation. Furthermore, safety remains a critical concern. As observed with rotenone, non-selective inhibition of complex I carries a risk of neurotoxicity. Therefore, future medicinal chemistry efforts must focus on structural optimization to improve stability and on the development of mitochondria-targeted delivery systems to enhance the therapeutic index.

Looking forward, integrating complex I inhibitors into combinatorial regimens represents a rational translational strategy. Due to the metabolic plasticity of cancer cells, monotherapy may lead to compensatory upregulation of glycolysis. Consequently, combining these agents with glycolytic inhibitors, immune checkpoint blockers, or standard chemotherapeutics offers a method to limit metabolic compensation, thereby enhancing therapeutic efficacy.

To advance the clinical application of these agents, a precision medicine approach is required. Future studies should focus on identifying predictive biomarkers, such as NQO1 expression or specific metabolic dependencies, to identify patients most likely to respond. Through the convergence of chemical biology, drug delivery, and patient stratification, natural products targeting mitochondrial complex I have the potential to be developed from experimental tools into effective clinical therapies.
